# Fluorescent β-Blockers as Tools to Study Presynaptic Mechanisms of Neurosecretion

**DOI:** 10.3390/ph4050713

**Published:** 2011-04-28

**Authors:** Beatriz Beltran, Romen Carrillo, Tomas Martin, Victor S. Martin, Jose D. Machado, Ricardo Borges

**Affiliations:** 1 Institute of BioOrganic “Antonio Gonzalez”, University of La Laguna, La Laguna 38071, Tenerife, Spain; E-Mails: bbeltran@ull.es (B.B.); vmartin@ull.es (V.S.M); 2 Institute de Natural Products and Agrobiology, Spanish National Research Council (CSIC), La Laguna 38071, Tenerife, Spain; E-Mails: rcarrillo@ipna.csic.es (R.C.); tmartin@ipna.csic.es (T.M.); 3 Unit of Pharmacology, Medical School, University of La Laguna, La Laguna 38071, Tenerife, Spain; E-Mail: jdmacha@ull.es (J.D.M)

**Keywords:** exocytosis, false neurotransmitter, secretory vesicle, TIRF

## Abstract

Several, if not all adrenergic β-blockers (β-Bs), accumulate progressively inside secretory vesicles in a time- and concentration-dependent manner, and could be considered to be false neurotransmitters. This transmitter effect is most likely unrelated to their ability to block adrenergic receptors, but it could explain the delay in lowering arterial pressure in hypertensive patients. We have developed a new drug to monitor the accumulation of β-Bs inside living cells, RCTM-3, which fluoresces in the visible spectrum. Here we describe the procedure to synthesize this new compound, as well as its fluorescent properties, pharmacological profile and its accumulation inside the secretory vesicles of PC12 cells.

## 1. Introduction

The use of radiolabeled molecules has demonstrated that adrenergic β-blockers (β-Bs) are accumulated and secreted by chromaffin cells [1,2]. Moreover, we recently demonstrated that β-Bs could exert their delayed effects as antihypertensive agents through a mechanism that is not directly related to their capacity as blockers of β-receptors but rather, to their progressive accumulation in secretory vesicles of sympathetic cells [3]. Other classical drugs like amphetamines [4], tyramine [5] or hydralazine [6] can also be taken up by secretory vesicles, thereby displacing the natural neurotransmitter. Such substances sharing this characteristic are usually termed as false neurotransmitters. Indeed, when the material accumulated possesses a specific activity, as occurs with β-Bs, co-secretion causes the pharmacological antagonism of catecholamines at the synaptic junction.

Although most of the commercially available β-Bs fluoresce, their excitatory spectra are in the ultraviolet range, which means they are of little use for studies of subcellular localization. For instance, we can detect the release of labetalol, the most fluorescent commercially available β-B (excitation λ335/emission λ420 nm), yet its accumulation inside cells cannot be monitored at these wavelengths as they promote autofluorescence of the cell when observed by epi-fluorescence microscopy [3]. In addition to classical tools like acridine orange [7] or quinacrine [8], new fluorescent false neurotransmitters have been used to monitor the accumulation of amines in secretory vesicles [9]. Thus, to demonstrate that β-Bs are effectively accumulated into secretory vesicles, molecules that fluoresce strongly in the visible spectrum while maintaining their β-blocking activity are required.

Here, we describe the synthesis of several β-Bs based on modifications to propranolol, the prototypic β-B, as well as the characterization of their fluorescent properties, and of their α- and β–adrenergic blocking activity.

## 2. Results and Discussion

We used propranolol, a prototypic β-B, as the template for our molecular design. As the side chain of antagonists is similar or identical to that of the isoprenaline agonist, the initial intention was to simply modify propranolol by adding an extra aromatic ring and forming an anthracene (compound RCTM-1, Table 1). RCTM-1 fluoresced strongly and had moderate blocking activity. However, the drug was very unstable and was rapidly hydrolyzed. Thus, we developed other compounds with a view to stabilizing the molecule by adding an extra aromatic ring (RCTM-2 and -6), by linking the side chain to the central aromatic rings (RCTM-3, -4, -5 and -7) and/or by modifying the substituent of the rings (RTCM-3 and -7).

When these compounds were analyzed, the increase in the size and rigidity of the aromatic side provoked a loss in the blocking capacity and on occasion, in fluorescent yield (Table 1). Only RTCM-3 (Pat. P200801297) possessed sufficient blocking potency (IC_50_ 14 μM) and fluorescent excitation in the visible range (Table 1). As other well known β-Bs possess α-blocking activity (labetalol, carvedilol), we also assessed the effects of the RCTM's on α-adrenergic receptors in rat aorta rings pre-contracted with noradrenaline. As expected, none of the compounds tested affected the vascular contractile response triggered by noradrenaline (Table 1). Accordingly, we centered our studies on RTCM-3.

After overexpression of neuropeptide Y (NPY) coupled to the enhanced green fluorescent protein (NPY-EGFP) we could observe a pattern typical of a vesicular distribution (Figure 2B). Neuropeptide Y is a well-documented vesicular peptide that is selectively packaged into secretory organelles [10,11], and the sorting of this protein to large dense core vesicles is only observed in newly synthesized vesicles as old granules do not fluorescence. When PC12 cells were incubated for 10 min with RCTM-3 (10 μM), a similar typical vesicular pattern was also observed (Figure 2A). When both images from the same cell were merged, 67 ± 1.3% of RTCM-3 labeling co-localized with NPY-EGFP (286 vesicles from 8 cells, Figure 2C), and 86.2 ± 1.3% of the RTCM-3 pixels were co-localized with those of NPY-EGFP. The LDCV that contained NPY-EGFP but not RCTM-3 fluorescence probably corresponded to immature vesicles that had not been acidified, while the RCTM-3 labeled vesicles with no NPY are likely to be old vesicles formed prior to NPY-EGFP transfection. When adrenal chromaffin cells were used instead of PC12, the vesicles labeled with RCTM-3 were larger than the protein labeled vesicles, indicating that a higher proportion of ‘old’ vesicles is present as opposed to newly synthesized ones (not shown).

**Figure 1:**
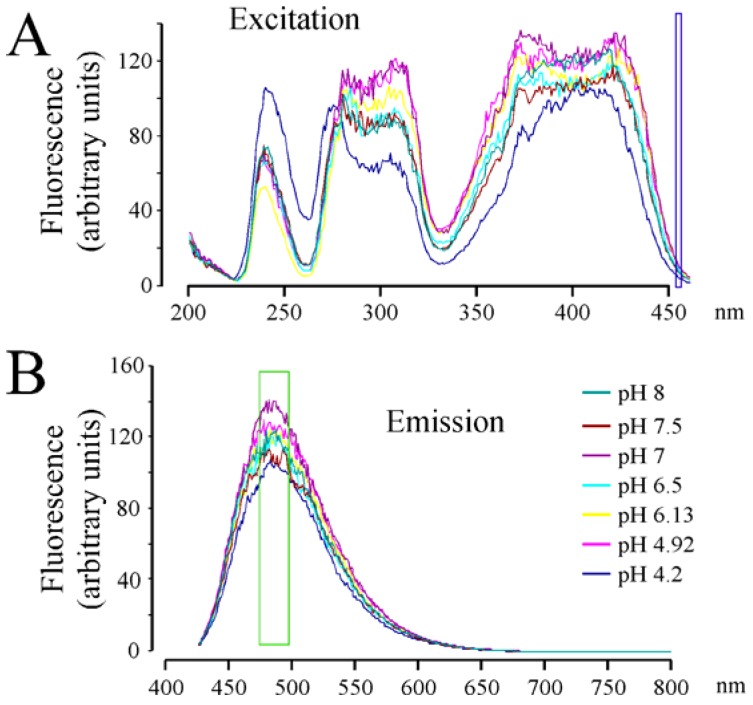
Fluorescent spectra of RCTM-3. (**A**). Excitation spectra showing three peaks at 240, 320 and 425 nm. The blue rectangle identifies the portion of the spectra illuminated by the laser used for TIRF microscopy; (**B**). Emission spectra, the green rectangle indicates the portion of the spectra selected by the band-pass filter used for TIRF microscopy. The colored lines indicate the fluorescent spectra obtained at different pH's.

**Figure 2:**
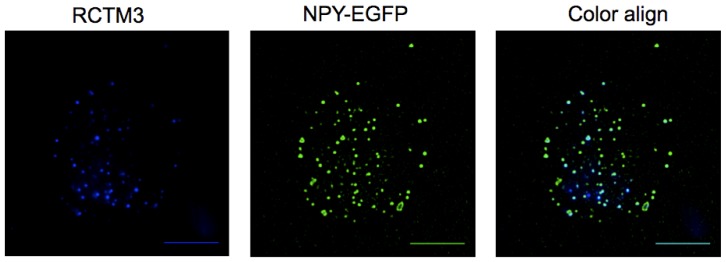
Intracellular location of RTCM-3 in a PC12 cell. (**A**). TIRFM images obtained from eight PC12 cells incubated for 10 min with RTCM-3 (10 μM); and (**B**). transfected with human pro-NPY tagged with EGFP (NPY-EGFP) Panel (**C**) shows a merged image of the other two images where coincident pixels are yellow (Bar, 10 μm).

When the fluorescent spectrum of RCTM-3 was studied (Figure 1), three peaks were evident in the excitation spectra and one in the emission spectra, characteristics that made it ideal to use in living cells that do not autofluorescence when excited at 425 nm. We also explored the pH dependence of this fluorescence as secretory vesicles have a pH of about 5.5 [8]. However, we only observed important changes in the excitation spectra of RCTM-3 at very acid pHs (e.g., pH 4.2), which is unlikely to be reached within vesicles (Figure 1). Nevertheless, the strong fluorescence of this compound means that even the spectral position of our laser line can be used (460 nm, blue square in Figure 1A). Despite these unfavorable conditions, the accumulation of RCTM-3 can be monitored by TIRF microscopy.

**Figure 3:**
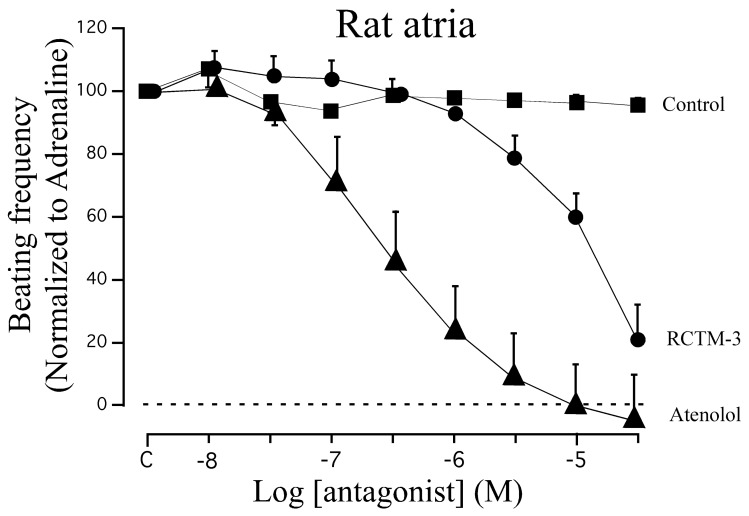
Inhibitory effects of RCTM-3 on isolated rat atria. Once stabilized, the preparations were stimulated with adrenaline (300 nM) and the frequency of beating was measured and normalized to 100%. Cumulative concentrations of no drug (control), RCTM-3 or atenolol were added to the organ bath. Data show the means ± SEM from 8–10 different experiments.

β-blocker activity has classically been assayed in isolated rat atria. Although this preparation beats spontaneously at ≈200 b.p.m., β-adrenergic agonists like adrenaline can increase the beating frequency to about 300 b.p.m. We demonstrated that adrenaline (300 nM) can maintain this frequency of beating for at least 20 min, sufficient time to test eight cumulative concentrations of β-Bs (10^−8^−3 × 10^−5^ M: Figure 3). When compared with the β_1_ cardioselective blocker atenolol, RCTM-3 showed moderate blocking activity (IC_50_ 14.1 *vs.* 0.28 μM) but no action on rat aorta rings pre-contracted with phenylephrine (Table 1). This response is typical of β-Bs that lack α activity, usually the initial test in screening procedures.

To reveal the presence of β-Bs within subcellular structures such as PC12 secretory vesicles, we performed experiments using total internal reflection fluorescence microscopy (TIRFM). PC12 cells were transfected with a NPY-EGFP plasmid and 48 h later they were incubated with RCTM-3 (10 μM) for 10 min. Double labeled structures were typically seen, suggesting the co-localization of EGFP (green) and RCTM-3 (blue: Figure 3).

Over twenty years ago the unexpected accumulation and secretion of radiolabeled β-Bs in chromaffin cells was demonstrated, as well as the increase of radioactivity in the bathing media when secretion is stimulated [1]. Recently, we also showed that the accumulation of β-Bs like atenolol, labetalol and propranolol (false neurotransmitters) has a significant effect on the quantum release of adrenaline [3]. Likewise, we demonstrated that the β-B labetalol was co-liberated with catecholamines. However, at that time we could not demonstrate the selective accumulation of β-Bs inside secretory vesicles as the fluorescence of the available β-Bs was largely affected by cell autofluorescence. The availability of RCTM-3 now provides direct evidence that these organelles do indeed accumulate β-Bs. Thus, the use of these fluorescent derivatives opens new perspectives for studying the interaction of drugs that affect secretory organelles at the subcellular level.

## 3. Experimental

### 3.1. General

^1^H-NMR spectra were recorded at 400 or 300 MHz, whereas ^13^C-NMR spectra were recorded at 75 or 100 MHz. The chemical shifts are reported relative to internal Me_4_Si and the coupling constants are given in Hz. Chromatography was performed on 60 Å and 0.2–0.5 mm silica gel columns and the compounds were visualized by use of UV light, 2.5% phosphomolybdic acid in ethanol, or vanillin with acetic and sulfuric acid in ethanol with heating. All solvents were purified by standard techniques. Reactions requiring anhydrous conditions were performed under nitrogen and anhydrous magnesium sulfate was used to dry the solutions.

#### 1-(Anthracen-1-yloxy)-3-(isopropylamino)propan-2-ol (**RCTM-1**, Scheme 1)

NaH (124 mg, 3.1 mmol, 60% oil dispersion) was added to a solution of anthracen-1-ol (0.5 g, 2.6 mmol) in dry tetrahydrofuran THF (26 mL) at room temperature. The mixture was stirred for 10 min and epibromohydrine (0.33 mL, 3.9 mmol) and tetrabutylammonium iodide (0.1 g, 0.26 mmol) were then added. The reaction was stirred overnight, after which TLC showed that the starting material had disappeared. The reaction mixture was then diluted with Et_2_O, and the mixture was washed with an aqueous saturated NH_4_Cl solution, dried, filtered, concentrated and purified by silica gel flash-chromatography, yielding the glycidyl ether (456 mg, 70% yield) as an oil. The glycidyl ether (0.4 g, 1.6 mmol) was mixed with isopropylamine (0.34 mL, 4.0 mmol) in EtOH (2 mL/mmol), refluxed for 3 h and left stirring at room temperature overnight. The solvent was evaporated and the crude mixture was purified by silica gel flash-chromatography, to yield **RCTM-1** (470 mg, 95s% yield) as a light brown solid: m.p. 127–129 °C; ^1^H-NMR (CDCl_3_, 300 MHz) δ 1.34 (m, 6H), 3.19 (m, 3H), 4.12 (dd, *J* = 4.9, 9.8 Hz, 1H), 4.20 (dd, *J* = 4.9, 9.8 Hz, 1H), 4.65 (m, 1H), 5.56 (br s, 2H), 6.55 (d, *J* = 7.5 Hz, 1H), 7.25 (d, *J* = 8.7 Hz, 1H), 7.43 (m, 2H), 7.54 (d, *J* = 8.4 Hz, 1H), 7.93 (d, *J* = 7.2 Hz, 1H), 8.08 (d, *J* = 8.7 Hz, 1H), 8.31 (s, 1H), 8.86 (s, 1H); ^13^C-NMR (CDCl_3_, 75 MHz) δ 20.4 (q), 20.5 (q), 48.6 (t), 50.3 (d), 66.8 (d), 69.8 (t), 102.4 (d), 120.7 (d), 124.5 (s), 124.9 (d), 125.0 (d), 125.5 (d), 127.6 (d), 128.6 (d), 131.0 (s), 131.7 (s), 132.3 (s), 153.8 (s); HRMS (ESI): *m/z*: calculated for C_20_H_24_NO_2_ [M^+^ + H]: 310.1807, found: 310.1808.

#### 1-(Isopropylamino)-3-(pyren-1-ylmethoxy)propan-2-ol (**RCTM-2**, Scheme 2)

The same procedure as that used above to obtain **RCTM-1** was applied to 1-pyrenemethanol on a 200 mg (0.86 mmol) scale, yielding the amino alcohol **RCTM-2** (260 mg, 87% yield in two steps) as a pale yellow solid: m.p. 79–84 °C; ^1^H-NMR (CDCl_3_, 300 MHz) δ 1.02 (m, 6H), 2.63 (dd, *J* = 8.1, 11.9 Hz, 1H), 2.75 (m, 2H), 3.23 (br s, 2H), 3.59 (m, 2H), 3.95 (m, 1H), 5.23 (s, 2H), 7.99 (m, 4H), 8.14 (m, 4H), 8.34 (d, *J* = 9.2 Hz, 1H); ^13^C-NMR (CDCl_3_, 75 MHz) δ 22.0 (q), 22.1 (q), 48.9 (d), 49.0 (t), 68.4 (d), 71.8 (t), 72.5 (t), 123.1 (d), 124.2 (d), 124.5 (s), 124.7 (s), 125.0 (d), 125.7 (d), 126.9 (d), 127.1 (d), 127.3 (d), 127.6 (d), 129.2 (s), 130.5 (s), 130.8 (s), 131.0 (s), 131.2 (s); HRMS (ESI): *m/z*: calculated for C_23_H_26_NO_2_ [M^+^ + H]: 348.1964, found: 348.1959.

#### 10-(2-Hydroxy-3-(isopropylamino)propyl)-4-methoxyacridin-9(10H)-one (**RCTM-3**, Scheme 3)

The same procedure as that used above to obtain **RCTM-1** was applied to 9-hydroxy-4-methoxyacridine on a 200 mg (0.89 mmol) scale, yielding the amino alcohol **RCTM-3** (190 mg, 63% yield in two steps) as a yellow solid: m.p. 178–182 °C; ^1^H-NMR (CDCl_3_, 400 MHz) δ 0.92 (m, 6H), 2.34 (dd, *J* = 9.0, 11.7 Hz, 1H), 2.53 (dd, *J* = 2.8, 11.8 Hz, 1H), 2.67 (m, 1H), 3.80 (s, 3H), 4.05 (br s, 3H), 4.48 (m, 2H), 6.96 (m, 3H), 7.42 (dd, *J* = 7.2, 7.2 Hz, 1H), 7.81 (m, 2H), 8.14 (d, *J* = 7.9 Hz, 1H); ^13^C-NMR (CDCl_3_, 100 MHz) δ 22.0 (q), 22.0 (q), 49.0 (d), 49.8 (t), 55.1 (t), 56.3 (q), 69.1 (d), 115.4 (d), 117.9 (d), 119.2 (d), 121.4 (d), 121.9 (d), 122.5 (s), 125.2 (s), 126.5 (d), 133.3 (d), 134.6 (s), 145.1 (s), 149.5 (s), 178.1 (s); IR (CHCl_3_) (cm^−l^) 3327, 3045, 2970, 1631, 1602, 1493, 1362, 1249; HRMS (ESI): *m/z:* calculated for C_20_H_25_N_2_O_3_ [M^+^ + H]: 341.1865, found: 341.1867.

#### 1-(Isopropylamino)-3-(phenanthren-9-yloxy)propan-2-ol (**RCTM-4**, Scheme 4)

The same procedure as that used above to obtain **RCTM-1** was applied to 9-phenanthrol on a 200 mg (1.03 mmol) scale, yielding the amino alcohol **RCTM-4** (203 mg, 64 % yield in two steps). The synthesis, and the spectroscopic and physical data for this compound have been described previously [12].

#### 1-(Anthracen-9-ylmethoxy)-3-(isopropylamino)propan-2-ol (**RCTM-5**, Scheme 5)

The same procedure as that used above to obtain **RCTM-1** was applied to 9-anthracenemethanol on a 200 mg (0.96 mmol) scale, yielding the amino alcohol **RCTM-5** (203 mg, 64% yield in two steps) as a light brown solid: m.p. 82–84 °C; ^1^H-NMR (CDCl_3_, 400 MHz) δ 0.98 (m, 6H), 2.53 (dd, *J* = 8.1, 11.8 Hz, 1H), 2.53 (dd, *J* = 3.7, 12.1Hz, 1H), 2.69 (m, 1H), 3.23 (br s, 2H), 3.62 (m, 2H), 3.87 (m, 1H), 5.46 (d, *J* = 11.6 Hz, 1H), 5.49 (d, *J* = 11.6 Hz, 1H), 7.47 (dd, *J* = 7.8, 7.8 Hz, 2H), 7.55 (dd, *J* = 6.9, 6.9 Hz, 2H), 7.99 (d, *J* = 8.4 Hz, 2H), 8.37 (d, *J* = 8.8 Hz, 2H), 8.43 (s, 1H); ^13^C-NMR (CDCl_3_, 100 MHz) δ 22.5 (q), 48.9 (d), 49.4 (t), 65.3 (t), 68.8 (d), 72.9 (t), 124.3 (d), 125.0 (d), 126.3 (d), 128.4 (d), 128.6 (s), 129.0 (d), 131.0 (d), 131.4 (s); HRMS (ESI): *m/z*: calculated for C_21_H_26_NO_2_ [M^+^ + H]: 324.1964, found: 324.1963.

#### 1-(Isopropylamino)-3-(pyren-1-yloxy)propan-2-ol (**RCTM-6**, Scheme 6)

The same procedure as that used above to obtain **RCTM-1** was applied to 1-hydroxypyrene on a 45 mg (0.21 mmol) scale, although the first reaction was carried out at 50 °C, yielding the amino alcohol **RCTM-6** (64 mg, 91% yield in two steps) as a light yellow solid: m.p. 168–172 °C; ^1^H-NMR (CDCl_3_, 400 MHz) δ 1.49 (m, 6H), 3.38 (m, 2H), 3.55 (m, 1H), 4.15 (dd, *J* = 5.5, 9.6 Hz, 1H), 4.22 (dd, *J* = 4.5, 9.6 Hz, 1H), 4.95 (m, 1H), 7.02 (d, *J* = 8.5 Hz, 1H), 7.59 (d, *J* = 8.5 Hz, 1H), 7.67 (d, *J* = 9.0 Hz, 1H), 7.74 (d, *J* = 9.0 Hz, 1H), 7.85 (m, 3H), 7.97 (d, *J* = 7.5 Hz, 1H), 8.32 (d, *J* = 9.1 Hz, 1H); ^13^C-NMR (CDCl_3_, 100 MHz) δ 19.0 (q), 19.2 (q), 48.0 (t), 51.8 (d), 66.0 (d), 70.2 (t), 108.9 (d), 119.9 (s), 120.8 (d), 124.2 (d), 124.3 (d), 124.6 (s), 125.1 (d), 125.3 (d), 125.5 (s), 126.0 (d), 126.6 (d), 127.0 (d), 131.4 (d), 131.5 (d), 151.7 (s); HRMS (ESI): *m/z:* calculated for C_22_H_24_NO_2_ [M^+^ + H]: 334.1807, found: 334.1800.

#### 10-(2-Hydroxy-3-(isopropylamino)propyl)acridin-9(10H)-one (**RCTM-7**, Scheme 7)

The same procedure as that used above to obtain **RCTM-1** was applied to acridone on a 100 mg (0.51 mmol) scale, yielding the amino alcohol **RCTM-7** (58 mg, 64% yield in two steps) as yellow solid: m.p. 99–101 °C; ^1^H-NMR (CD_3_OD, 400 MHz) δ 1.21 (t, *J* = 6.2 Hz, 6H), 3.00 (dd, *J* = 9.0, 12.3 Hz, 1H), 3.09 (m, 2H), 4.38 (m, 1H), 4.51 (dd, *J* = 3.6, 16.0 Hz, 1H), 4.65 (dd, *J* = 8.8, 16.0 Hz, 1H), 7.24 (dd, *J* = 7.5, 7.5 Hz, 2H), 7.74 (m, 2H), 7.86 (m, 2H), 8.33 (dd, *J* = 1.7, 8.0 Hz, 2H); ^13^C-NMR (CD_3_OD, 100 MHz) δ 21.3 (q), 21.4 (q), 50.4 (t), 50.8 (t), 51.1 (d), 68.6 (d), 117.8 (d), 122.9 (d), 123.2 (s), 128.1 (d), 135.4 (d), 144.1 (s), 179.9 (s); IR (CHCl_3_) (cm^−l^) 3688, 3432, 3020, 1604, 1493; HRMS (ESI): *m/z*: calculated for C_19_H_23_N_2_O_2_ [M^+^ + H]: 311.1760, found: 311.1764.

#### Dansyl analogue of propanolol (**RCTM-8**, Scheme 8)

The synthesis for this compound was described previously by Atlas and Levitzki [13].

#### 1-(Anthracen-9-yloxy)-3-(isopropylamino)propan-2-ol (**RCTM-9**, Scheme 9)

The same procedure as that used above to obtain **RCTM-1** was applied to anthrone on a 200 mg (1.03 mmol) scale, yielding the amino alcohol **RCTM-9** (143 mg, 45% yield in two steps). The synthesis, and the spectroscopic and physical data for this compound have been described elsewhere [12].

#### 1-(9H-Fluoren-9-yloxy)-3-(isopropylamino)propan-2-ol (**RCTM-10**, Scheme 10)

The same procedure as that used above to obtain **RCTM-1** was applied to 9-hydroxyfluorene on a 200 mg (1.10 mmol) scale, yielding the amino alcohol **RCTM-10** (228 mg, 70% yield in two steps) as a light yellow solid: m.p. 83–84 °C; ^1^H-NMR (CDCl_3_, 400 MHz) δ 1.05 (m, 6H), 2.56 (m, 1H), 2.68 (m, 1H), 2.79 (m, 1H), 3.13 (m, 4H), 3.80 (m, 1 H), 5.65 (s, 1H), 7.29 (dd, *J* = 7.5, 7.5 Hz, 2H), 7.37 (dd, *J* = 7.2, 7.2 Hz, 2H), 7.58 (d, *J* = 7.2 Hz, 2H), 7.64 (d, *J* = 7.2 Hz, 2H); ^13^C-NMR (CDCl_3_, 100 MHz) δ 23.0 (q), 49.4 (d), 49.8 (t), 67.6 (t), 69.3 (d), 81.2 (d), 120.4 (d), 125.9 (d), 128.0 (d), 128.0 (d), 129.5 (d), 141.3 (s), 142.9 (s); Anal. calculated for C_19_H_23_NO_2_ C 76.73, H 7.80, N 4.71, found: C 76.55, H 7.78, N 4.69.

#### 1-((5H-Dibenzo[a,d][7]annulen-5-yl)oxy)-3-(isopropylamino)propan-2-ol (**RCTM-11**, Scheme 11)

The same procedure as that used above to obtain **RCTM-1** was applied to dibenzosuberenol on a 500 mg (2.40 mmol) scale, yielding the amino alcohol **RCTM-11** (414 mg, 53% yield in two steps) as a white solid: m.p. 112–114 °C; ^1^H-NMR (DMSO-*d*_6_, 500 MHz, 363 K) δ 0.98 (d, *J* = 6.4 Hz, 6H), 2.53 (m, 1H), 2.66 (m, 1H), 2.72 (m, 1H), 2.98 (br s, 2H), 3.41 (s, 2H), 3.72 (s, 1H), 5.01 (br s, 1H), 7.12 (s, 2H), 7.29 (dd, *J* = 7.0, 7.0 Hz, 2H), 7.41 (m, 4H), 7.65 (m, 2H); ^13^C-NMR (DMSO-*d*_6_, 125 MHz, 363 K) δ 23.2 (q), 23.3 (q), 48.6 (d), 50.6 (t), 69.6 (d), 72.8 (t), 79.6 (d), 126.9 (d), 128.6 (d), 128.6 (d), 133.4 (s), 139.3 (s); Anal. calculated for C_21_H_25_NO_2_ C 77.98, H 7.79, N 4.33, found: C 78.14, H 7.64, N 4.28.

### 3.2. Fluorescence

Fluorescent spectra were obtained on a Cary Eclipse spectrofluorimeter (Varian Cary Eclipse, Agilent Technologies, Santa Clara, CA, USA) after dissolving the molecules in DMSO and then diluting them in saline buffer at the pH indicated.

### 3.3. Isolated Rat Atria and Aorta Rings

All animal procedures were performed in agreement with institutional and national guidelines and regulations and approved by the Ethical Committee of the University of La Laguna (res#49/2004). The organ baths were prepared as described elsewhere [14]. Briefly, animals were sacrificed by decapitation, their hearts were rapidly removed and the right atrium was placed in a 4 mL organ bath cup. A basal tension of 1 g was applied and the contractions monitored using an isometric transducer at a temperature of 37 °C in Krebs-bicarbonate solution (in mM: NaCl [119], KCl [4.7], MgSO_4_ [1.2], KH_2_PO_4_ [1.2], CaCl_2_ [2.5], NaHCO_3_ [25] and glucose [13]) continuously bubbled with 95% O_2_, 5% CO_2_ mixture to maintain the pH at 7.4.

Thoracic aortas were excised and cleaned of surrounding fat and connective tissues. Slices of ≈2 mm were cut carefully to avoid endothelial damage, and they were mounted in the organ bath as described above using Krebs-bicarbonate solution and applying a basal tension of 1 g. The data were sampled at 400 Hz using Chart for Macintosh and Powerlab (ADInstruments, Dunedin, New Zealand), and the data was quantified and analyzed using Graphpad Prism 5 software (La Jolla, CA, USA).

### 3.4. Neuropeptide Y Construct

The human pro-neuropeptide Y construct fused to EGFP (NPY-EGFP) was kindly provided by W. Almers (Vollum Institute, Oregon Health & Science University) [10,11].

### 3.5. PC12 Cell Culture and Transfection

PC12 cells were maintained in 75-mL flasks at 37 °C and 5% CO_2_ using RPMI 1640 supplemented with 10% fetal calf serum and 5% horse serum. For TIRFM experiments, PC12 cells in suspension were nucleofected using a Nucleofector device and following the manufacturer's protocol. Briefly, 1 × 10^6^ cells were dissociated and re-suspended in 100 μL of the specific Amaxa kit solution (Amaxa GmbH, Koeln, Germany) containing NPY-EGFP (2 μg) cDNA, and they were transferred to an electroporation cuvette. Cells were electroporated using the U29 cell specific program and they were then plated at an approximate density of 2.5 × 10^6^ cells on poly-L-lysine-coated 18 mm diameter coverslips (*n* = 1.518; Warner Instruments, Hamdem, CT), which were placed in 6-well plates. The cells were incubated for 5 h in RPMI without sera and antibiotics, after which the medium was supplemented with 10% FBS and the cells were allowed to adhere overnight. The cells were used 48 h later.

### 3.6. Fluorescence Microscopy

Cells were visualized on an inverted microscope (Zeiss 200M, Carl Zeiss, Jena, Germany) using a 1.45 NA objective (alpha Fluar, 100X/1.45, Zeiss). The objective was coupled to the coverslip using an immersion fluid (n488 = 1.518; Zeiss). For evanescent field illumination, the expanded beam of an argon ion laser (Lasos, Lasertechnik GmbH, Germany) was band-pass filtered and used to selectively excite different fluorophores. Different filters were used for each fluorophor analyzed and the beam was focused off-axis position at the rear focal plane of the objective. When passing through the coverslip, light underwent total internal reflection as it struck the interface between the glass and the solution or cell at a glancing angle. Total internal reflection generates an evanescent field that declines exponentially as the distance from the interface increases, depending on the angle at which the light strikes the interface [15,16]. The images obtained were projected onto a CCD camera (AxioCam MRm, Zeiss) though a dichroic FT500 and band-pass filter (525/50 nm) for EGFP signals, or through a dichroic FT470 and band-pass filter (485/20 nm). Up to five cells were imaged on each coverslip.

### 3.7. Image Analysis

To determine the overlap between the different fluorescent molecules, evanescent field images were analyzed as described recently [17]. Briefly, the images were low-pass filtered using Metamorph (Molecular Devices, Sunnyvale, CA, USA), and a circle 10 pixels in diameter was plotted around each spot analyzed, as well as five further circles outside these spots that were used to calculate the local background. We then drew circles 10 pixels in diameter around the NPY-EGFP spots and overlayed these circles onto the image of the paired molecules at identical pixel locations. Finally, we determined whether the new circle contained a concentric fluorescent point within the 10 pixel circle to quantify the co-localization of the fluorophor. Circles were scored as positive if they contained a fluorescent spot and negative if they did not. Moreover, the co-localization was only scored as positive when the average fluorescence intensity was at least three times the standard deviation of the background. The percentage co-distribution was determined in single cells after random co-distribution subtraction, and the average values were calculated from the total number of cells analyzed.

## 4. Conclusions

We have developed a new fluorescent beta-adrenergic blocker with an excitation spectrum within visible wavelengths. This particular feature allows fluorescence microscopy to be used to observe how it accumulates inside secretory vesicles of PC12 cells. This new tool is of particular interest to label secretory vesicles, as well as to gain evidence that β-Bs can exert their delaying hypotensive effects through the displacement of natural neurotransmitters from sympathetic neurons.

## Figures and Tables

**Scheme 1:**
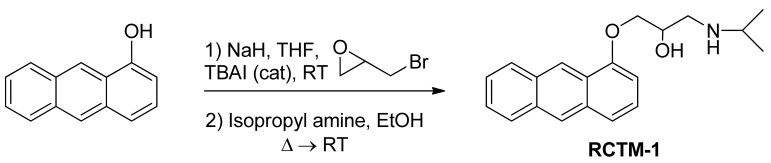
Preparation of **RCTM-1**.

**Scheme 2:**
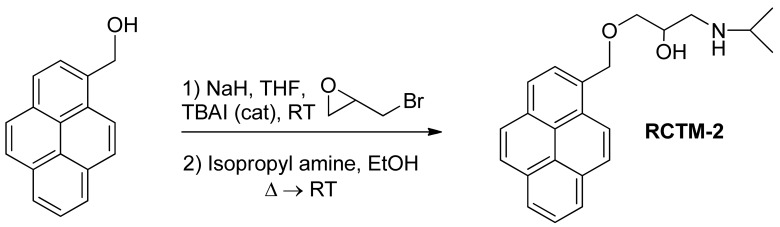
Preparation of **RCTM-2**.

**Scheme 3:**
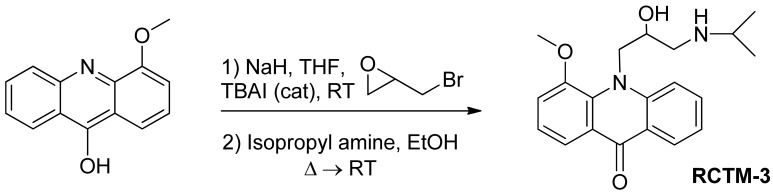
Preparation of **RCTM-3**.

**Scheme 4:**
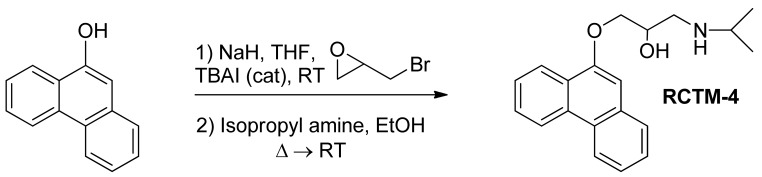
Preparation of **RCTM-4**.

**Scheme 5:**
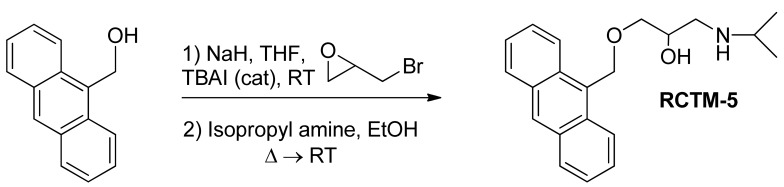
Preparation of **RCTM-5**.

**Scheme 6:**
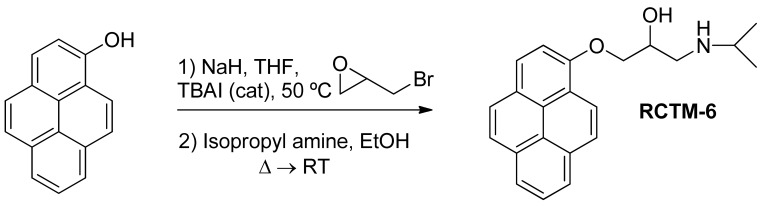
Preparation of **RCTM-6**.

**Scheme 7:**
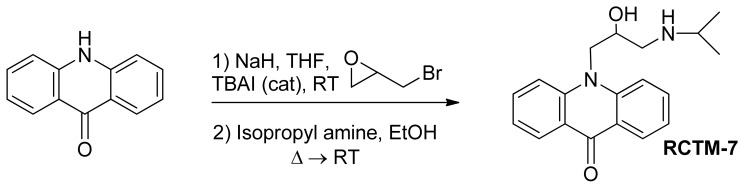
Preparation of **RCTM-7**.

**Scheme 8:**
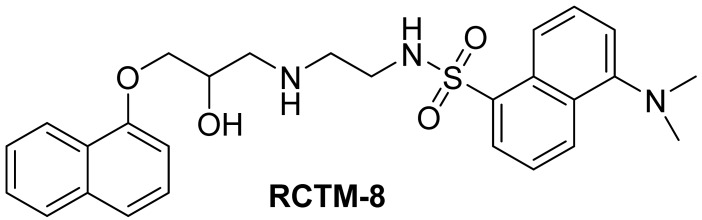
Preparation of **RCTM-8**.

**Scheme 9:**
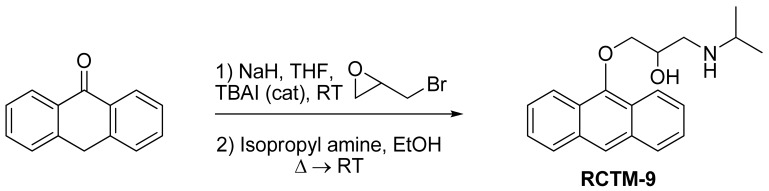
Preparation of **RCTM-9**.

**Scheme 10:**
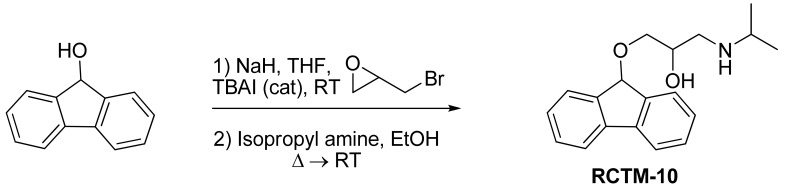
Preparation of **RCTM-10**.

**Scheme 11:**
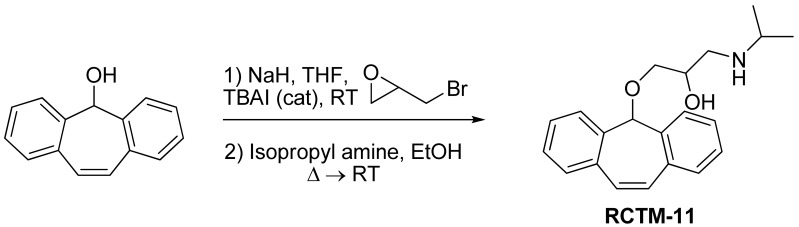
Preparation of **RCTM-11**.

**Table 1:** Chemical structures, EC50 in rat atria and aorta, as well as the fluorimetric properties of the new compounds.

**Compound**	**MW**	**Chemical structure**	**IC_50_ (rat atria) (μM)**	**IC_50_ (rat aorta) (μM)**	**Fluorescent profile λ (nm)**
RCTM-1	309.40	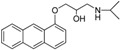	0.6	>30	365/435
RCTM-2	347.45	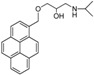	>30	>30	356/394
RCTM-3	340.41	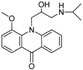	14.1	>30	425/480
RCTM-4	309.40	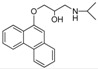	0.2	>30	350/376
RCTM-5	323.42	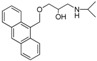	18.2	>30	346/420
RCTM-6	333.42	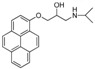	>30	>30	344/770
RCTM-7	310.39	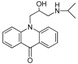	>30	>30	390/425
RCTM-8	493.61	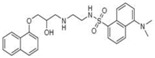	>30	n.d.	315/550
RCTM-9	309.40	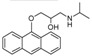	>30	n.d.	334/426
RCTM-10	297.39	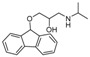	>30	n.d.	372/423
RCTM-11	323.43	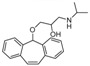	>30	n.d.	317/346

**n.d.** Activity not determined. **Fluorescent profile** in terms of max excitation/max emission wavelengths (in nm)
